# Prenatal Diagnosis of Cystic Hygroma related to a Deletion of 16q24.1 with Haploinsufficiency of FOXF1 and FOXC2 Genes

**DOI:** 10.1155/2012/490408

**Published:** 2012-08-28

**Authors:** Matthew J. Garabedian, Donna Wallerstein, Nubia Medina, James Byrne, Robert J. Wallerstein

**Affiliations:** ^1^Department of Obstetrics and Gynecology, Santa Clara Valley Medical Center, San Jose, CA 95128, USA; ^2^Department of Pediatrics and Genetics, Santa Clara Valley Medical Center, San Jose, CA 95128, USA

## Abstract

We report the prenatal diagnosis of cystic hygroma that was subsequently identified to have haploinsufficiency of the FOXF1 and FOXC2 genes via array comparative genomic hybridization (aCGH). Deletion o f these genes has previously neither been associated with cystic hygroma nor prenatally diagnosed. The FOX gene cluster is involved in cardiopulmonary development. This case expands the phenotypic spectrum o f abnormalities of the FOXF1 and FOXC2 genes, as it seems within the spectrum of function that disruption of the FOX gene cluster would lead to include abnormalities of prenatal onset. Identification of this association would not be possible with conventional karyotype or targeted aCGH. This case highlights the power of whole genomic aCGH to further delineate the etiology of birth defects.

## 1. Introduction

Cystic hygroma is a developmental anomaly that has a strong association with chromosomal abnormalities such as trisomy 21 and monosomy X. It is long established that prenatal diagnosis of chromosome abnormalities is recommended for the evaluation of such an affected fetus. Array comparative genomic hybridization technology (aCGH) allows for the detection of submicroscopic genomic alterations and is becoming a more standard analysis for the evaluation of pregnancies with anomalies. With this case, we illustrate how whole genome aCGH allows for the enhanced antenatal detection of genetic syndromes with the first report of antepartum detection of haploinsufficiency of the FOXF1 and FOXC2 genes. 

## 2. Case Presentation

**Figure 1:**
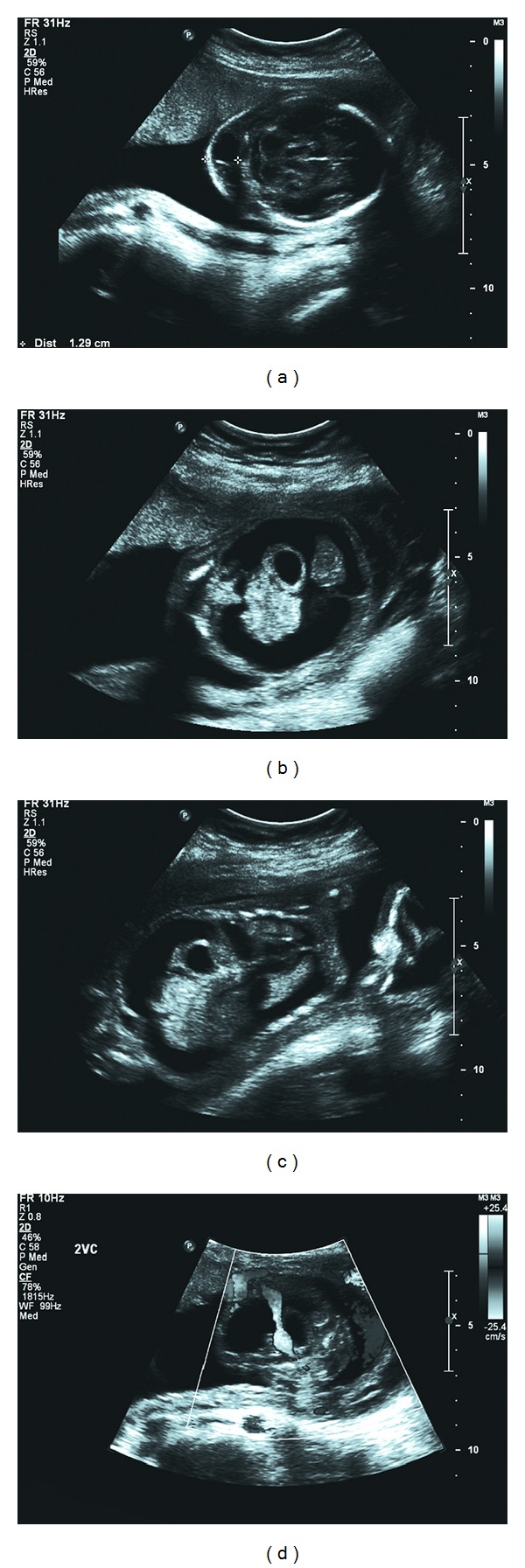
Ultrasound images of (a) septated cystic hygroma, (b) ascites with free floating stomach and small intestine, (c) ascites and pleural effusions (coronal view), and (d) single umbilical artery.

A 29-year-old Gravida 4 Para 3-0-0-3 woman presented at 18 weeks 5 days gestation for screening ultrasound. A septated cystic hygroma, fetal hydrops, and a single umbilical artery were noted (Figure 1). Genetic counselling was provided. Review of the obstetric and family history showed no reported cases of birth defects, mental retardation, consanguinity, or known genetic conditions. The patient and her husband were counselled regarding the dismal prognosis in the setting of fetal hydrops. 

Amniocentesis was performed with amniotic fluid sent for fluorescent in situ hybridization (FISH) analysis of trisomies of chromosomes 13, 18, 21, X, and Y, karyotype, and whole genome single nucleotide polymorphism (SNP-)based copy number microarray analysis targeting 2.695 million copy number and allele-specific genome sites was performed on cultured amniotic fluid cells.

FISH analysis and karyotype were normal (46 XY), and the patient elected to undergo termination of pregnancy, despite normal karyotype, given the severity of the fetal condition. The patient opted for a dilatation and evacuation (D&E) procedure. Preoperative ultrasound revealed a 21-week sized fetus with cystic hygroma and ascites. Intracardiac digoxin was administered the day prior to surgery. At 22 weeks gestation, the patient underwent D&E without complication. Pathologic evaluation of the fetus was limited to gross examination. Low set ears and soft tissue edema of the neck were noted. 

**Figure 2:**
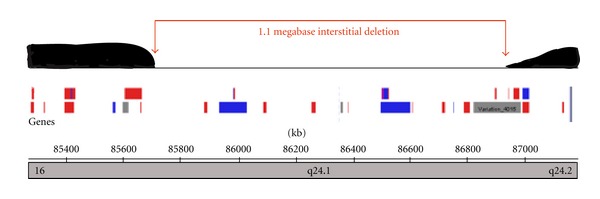
Array comparative genomic hybridization (aCGH) map of 16q24.1 demonstrating 1.1 megabase interstitial deletion.

Results from the microarray became available after the termination of pregnancy and were consistent with a male fetus with a 1.1 megabase interstitial deletion of chromosome 16 at position 16q24.1 (16q24.1(85, 728, 812-86, 831, 579) x1) leading to haploinsufficiency of the genes FOXF1 and FOXC2 (Figure 2). This deletion was confirmed by bacterial artificial chromosome FISH.

## 3. Discussion

FOXF1 and FOXC2 are part of the forkhead gene family, which are transcription factors originally identified as potential tumor suppressor genes [1]. In the human genome, members of the FoxC, FoxF, FoxL1, and FoxQ1 gene families are involved in patterning early embryonic mesoderm. Deletions in 16q24.1 in the region encoding for the FOX gene cluster have been associated with multiple structural anomalies, including cardiopulmonary anomalies and congenital alveolar capillary dysplasia with misalignment of the pulmonary veins (ACD/MPV); (OMIM 265380) [2]. Antenatal detection has not previously been reported. Other genes in this deletion (e.g., MTHFSD, FOXL1, IRF8, COX4I1, COX4NB) have not been associated with any known phenotype.

ACD/MPV is characterized histologically by failure of formation and in-growth of alveolar capillaries, medial muscular thickening of small pulmonary arterioles with muscularization of the intra-acinar arterioles, thickened alveolar walls, and anomalously situated pulmonary veins running alongside pulmonary arterioles which share the same adventitial sheath. Less common features include a reduced number of alveoli and a patchy distribution of the histopathologic changes. The disorder is associated with persistent pulmonary hypertension of the neonate and is uniformly fatal within the newborn period [3, 4].

Disruption of the FOX gene cluster in association with cystic hygroma and fetal hydrops has not previously been reported. It would seem to be within the spectrum of gene function that disruption of this gene cluster would lead to this phenotype. Had autopsy been permitted by the family, our case would be strengthened if, for example, ACD/MPV were identified. We feel that this 1.1 Mb interstitial deletion of 16q24.1 is likely pathological, based upon an accepted algorithm for assessment of the significance of a novel copy number variant (CNV) [5]. Consequently, we recommend consideration of FOX gene cluster deletions and mutations in the differential diagnosis of fetal hydrops and cystic hygroma.

The prenatal diagnosis of cystic hygroma is well known to confer a high risk of poor pregnancy outcome, even with a normal karyotype [6, 7]. More than 90% of pregnancies affected by cystic hygroma will result in an abnormal outcome including chromosomal abnormalities, genetic syndromes, structural anomalies, spontaneous abortion, fetal loss, or neonatal death [8]. As such, a cystic hygroma is a clear indication for genetic counselling and prenatal diagnosis.

While chorionic villus sampling and amniocentesis are important components of prenatal diagnosis, only 50%–60% of fetuses with a cystic hygroma will have a demonstrable karyotypic abnormality [6–8]. The detection of chromosomal abnormalities can be increased by an additional 11.2% (95% confidence interval 5.7%–22.1%) with the utilization aCGH when structural anomalies, including cystic hygroma, are detected on ultrasound [9].When benign copy number variants (CNV) are excluded, aCGH will detect 5.2% (95% confidence interval 1.9%–13.9%) meaningful chromosomal anomalies that would not be detected by conventional karyotype [9]. Furthermore, aCGH has the benefits of providing results sooner than conventional karyotype and minimizing the risk of the development of an *in vitro* mosaic artifact from cell culture [10].

This case illustrates the role of aCGH in prenatal diagnosis. The results from the aCGH have improved counselling and, for another patient, may have influenced management. The high resolution of aCGH allows for precise genetic analysis and improved detection of anomalies in comparison to other forms of analysis [5, 9, 10]. The American College of Obstetricians and Gynecologists (ACOG) currently endorses the conventional karyotype as the primary modality for chromosomal analysis, with targeted aCGH as “an adjunct tool in prenatal cases with abnormal anatomic findings and normal karyotype” or in situations where it is not possible to obtain a karyotype [11]. The American College of Medical Genetics (ACMG) and the International Standard Cytogenomic Array (ISCA) Consortium provide guidelines for use of aCGH in postnatal evaluation without discussing its role in prenatal diagnosis [12, 13]. 

The value of whole genomic analysis is the ability to identify new genetic deletion or duplication syndromes [14]. Identifying new associations is not possible with the utilization of an array designed to detect known genetic pathology. While the interpretation of the finding of a novel CNV can be challenging and does require thoughtful, intelligent counseling; novel CNV may lead to the identification of new genetic syndromes [15].

While the exact role of aCGH may remain under discussion, aCGH is an important component in modern prenatal diagnosis. Whole genomic aCGH is allowed to be used to identify a new association between phenotype (septated cystic hygroma) and genotype (16q24.1 deletion) that would not have been made with conventionally karyotype or targeted aCGH. Further reports of this association would strengthen the possibility of prenatal diagnosis of this lethal condition. Utilization of whole genome aCGH in prenatal diagnosis is important to further understanding the genetic contribution to disease.
